# Induction of Apoptotic Effects of Antiproliferative Protein from the Seeds of *Borreria hispida* on Lung Cancer (A549) and Cervical Cancer (HeLa) Cell Lines

**DOI:** 10.1155/2014/179836

**Published:** 2014-01-30

**Authors:** S. Rupachandra, D. V. L. Sarada

**Affiliations:** Department of Biotechnology, School of Bioengineering, SRM University, Kattankulathur 603203, India

## Abstract

A 35 KDa protein referred to as F3 was purified from the seeds of *Borreria hispida* by precipitation with 80% ammonium sulphate and gel filtration on Sephadex G-100 column. RP-HPLC analysis of protein fraction (F3) on an analytical C-18 column produced a single peak, detected at 220 nm. F3 showed an apparent molecular weight of 35 KDa by SDS PAGE and MALDI-TOF-MS analyses. Peptide mass fingerprinting analysis of F3 showed the closest homology with the sequence of 1-aminocyclopropane-1-carboxylate deaminase of *Pyrococcus horikoshii*. The protein (F3) exhibited significant cytotoxic activity against lung (A549) and cervical (HeLa) cancer cells in a dose-dependent manner at concentrations ranging from 10 *µ*g to 1000 *µ*g/mL, as revealed by the MTT assay. Cell cycle analysis revealed the increased growth of sub-G0 population in both cell lines exposed to a concentration of 1000 *µ*g/mL of protein fraction F3 as examined from flow cytometry. This is the first report of a protein from the seeds of *Borreria hispida* with antiproliferative and apoptotic activity in lung (A549) and cervical (HeLa) cancer cells.

## 1. Introduction

Cancer is the largest single cause of death in men and women, and chemoprevention has been a promising anticancer approach aimed at reducing the morbidity and mortality of cancer by delaying the process of carcinogenesis. Among many recent advances in cancer chemotherapy, plant natural products play an important role in having contributed considerably to approximately 60% of available cancer chemotherapeutic drugs [1]. A new strategy in cancer therapy is to induce the apoptosis in tumor cells which is characterized by early activation of endogenous proteases, cell shrinkage, membrane blebbing, and DNA fragmentation [2].

Plant-derived compounds and their semisynthetic and synthetic analogs serve as major source of pharmaceuticals for human diseases [3]. One of the most active areas of research in the field of cancer therapy is the search for plant proteins with potent cytotoxic activity and low toxicity with varied mechanisms of action on tumors. Plant seeds are an enormously rich source of proteins with the potential to be developed as anticancer agents, for example, Violaceae [4], Rubiaceae [5], and Cucurbitaceae [6] families and certain marine plants [7].

*B. hispida* (Rubiaceae) is a promising medicinal plant which is widely used in folk medicine to treat fever due to primary complex, ulcer, and glandular swellings [8]. Ethnobotanically, *B. hispida *has been used as therapeutic agent in the treatment of various pathological conditions. It is used as an antieczemic and antibacterial and also used to treat cardiovascular disorders [9, 10]. The methanolic extract of seeds of *B. hispida* inhibited the growth of human lung carcinoma (A549) and breast carcinoma (MCF-7) cell lines [11]. The aim of this study is to isolate and purify the proteins exhibiting cytotoxic activity from the seeds of *Borreria hispida*. The significant effect of purified protein on cell proliferation and cell cycle arrest of lung (A549) and cervical (HeLa) cancer cell lines is also investigated.

## 2. Experimental

### 2.1. Materials

Seeds of *B. hispida* were collected and authenticated from the Plant Anatomy Research Centre, Chennai. All the reagents and chemicals were purchased from Sigma Aldrich. Buffers used for FPLC analysis were of analytical grade. Tumour cell lines, A549 (human lung adenocarcinoma epithelial cell line), and HeLa (human cervical adenocarcinoma epithelial cell line) were purchased from NCCS, Pune.

### 2.2. Protein Extraction

Seeds of *B. hispida* were washed with distilled water and shade dried. The dried seeds were ground to fine powder and the proteins were extracted with extraction buffer [12] consisting of 10 mM Na_2_ HPO_4_, 15 mM NaH_2_PO_4_, 10 mM KCl, and 2 mM EDTA (pH 7.0) by constant stirring overnight at 4°C. The crude protein extract was filtered using the muslin cloth and centrifuged at 10,000 rpm for 10 minutes. Proteins were precipitated from the crude supernatant using ammonium sulphate up to 80% saturation, overnight at 4°C. The precipitated proteins were collected by centrifugation at 12,000 rpm for 20 min. The protein concentration was determined according to Lowry et al. (1951) [13], using bovine serum albumin as standard.

### 2.3. Purification of Proteins

The precipitated protein fractions obtained by 80% ammonium sulphate saturation were applied onto Fast Protein Liquid chromatography (Akta purifier GE Healthcare) using Sephadex G-100 gel filtration column (1.5 × 50 cm) equilibrated with 50 mM Tris-HCl buffer (pH 7.5). The protein fractions were eluted using the same buffer at a flow rate of 0.1 mL/min and detected at 280 nm [14]. 2 mL fractions were collected and screened for cytotoxic activity against the selected cancer cell lines. The protein fraction exhibiting increased cytotoxic activity warranted further studies.

### 2.4.  RP-HPLC Analysis

The purity of protein fraction F3 which showed potent cytotoxic activity was tested using analytical reverse phase-HPLC- C_18_ column (250 × 4.6 mm, 5 *μ*m, 300 Å), with solution A being 0.1% TFA and solution B being acetonitrile (90%) as the mobile phase at a flow rate of 1 mL/min, and detected at 220 nm [15]. 

### 2.5. SDS-PAGE Analysis

**Figure 1:**
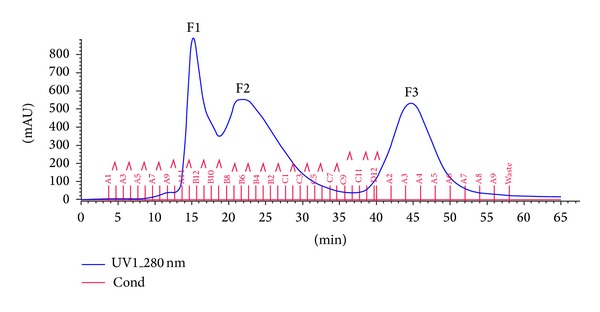
Size exclusion (Sephadex G-100) chromatogram of the protein fractions obtained from 80% ammonium sulphate precipitation from the seeds of *B. hispida*.

12% SDS-PAGE analysis of active protein fraction F3 was performed according to Laemmli et al. [16]. About 20 *μ*g/mL of F3 fraction was loaded in Lane 1 (Figure 1) and 20 *μ*g/mL of protein marker with the molecular weight in the range of 14.3 KDa–66 KDa was loaded in Lane 2. Electrophoresis was run at 100 V for 2 hrs. The protein bands were visualized using Coomassie Brilliant Blue (CBB) R-250 [17].

### 2.6. Identification of Protein Fraction F3 by MALDI-TOF Mass Spectrometry

The gel band of F3 was excised from SDS-PAGE and the tryptic digests were analysed using MALDI-TOF mass spectrometry which acts as a platform to generate the molecular mass of purified F3 protein [18]. In proteomics research, trypsin is commonly used for protein digestion to produce peptides with molecular masses in the optimal range for MS analysis. Prior to tryptic digestion, purified F3 protein was reduced and alkylated with tributylphosphine (TBP) and 15 mM iodoacetamide, respectively. About 2 *μ*g trypsin pre-dissolved in 1 mM HCl was added to F3 and digested for 12–18 hr at 37°C. An equal volume of cyano-4-hydrocinnamic acid solution (10 mg/mL CHCA in 70% ACN, 0.03% TFA) was added to the trypsinized protein fraction F3. MALDI-TOF spectrum depicts the fragmentation of F3 in to various peptides. Peptide mass fingerprinting (PMF) was carried out to examine the sequence of the peptide fragments of F3 using SWISS-PROT sequence database.

### 2.7. Evaluation of Cytotoxic Activity of Protein Fractions

The isolated protein fractions were tested for cytotoxic activity against lung (A549) and cervical (HeLa) cancer cell lines. A549 and HeLa cells were cultured in Dulbecco's modified eagle medium (GIBCO), supplemented with 10% fetal bovine serum (GIBCO) and 1% antibiotic antimycotic solution at 37°C in a humidified atmosphere of 5% CO_2_. A549 and HeLa cells were separately seeded at densities of 3500 and 3000 cells/well, respectively, in 96-well microtitre plates in a total volume of 200 *μ*L. The protein fractions were added at concentrations ranging from 10 *μ*g to 1000 *μ*g and cells were incubated. After 24 hours, the cellular morphology was observed using a phase contrast microscope (Leica, Wetzlar, Germany). MTT assay [19] was performed to evaluate the cytotoxic activity of the protein fractions. To each of the wells, 50 *μ*L (5 mg/mL) of 0.5% MTT was added and incubated for 4 h. The formazan crystals formed were dissolved in dimethyl sulfoxide (DMSO) and the absorbance was read at 570 nm using a microplate reader (Bio-Rad, CA). Wells without protein fractions served as blank. Doxorubicin (Sigma) was used as positive control. Percentage inhibition of cell viability was determined by using the formula *A*_control_ − *A*_treatment_/*A*_control_  × 100.

### 2.8. Cell Cycle Analysis by Flow Cytometry

A549 and HeLa cells were separately seeded in 6-well culture plates at a density of 0.3 × 10^6^ cells/well and allowed to attach to the plates by incubating at 37°C, 5% CO_2_ in an incubator for 24 h. The cells were treated with F3 at the concentration of 1000 *μ*g/mL using fresh media and incubated for 24, and 48 h for A549 and HeLa cells, respectively. The cells were trypsinized, washed with media, and centrifuged at 1000 rpm for 5 min at 4°C. The supernatant was discarded and cells were suspended in 300 *μ*L of PBS and 700 *μ*L of ethanol. The contents were kept at 4°C and then processed for cell cycle analysis by flow cytometry [20]. The cells were washed and centrifuged at 1500 rpm for 10 min at 4°C. The supernatant obtained was discarded and cells were resuspended in 600 *μ*L of PBS containing 0.5% Triton X-100 and 20 *μ*g RNase and incubated at room temperature for 1 h. To the cells, 24 *μ*L (40 *μ*g/mL) of propidium iodide (1 mg/mL stock) was added and again incubated at room temperature for 45 min in dark. The cells were detected in a flow cytometer (FACSCalibur, Beckton Dickinson) equipped with an air cooled argon laser providing 15 mW at 488 nm (blue laser) with standard filter setup. Ten thousand events were acquired and the percentage of cells in each phase of the cell cycle was analyzed using CellQuest Pro software (Becton Dickinson, USA).

### 2.9. Statistical Analysis

Data for *in vitro* experiments are expressed as mean ± S.E.M. Comparisons between treated groups and control were performed with Dunnett's Multiple Comparison Test with *P* < .0001, *P* < 0.001, and *P* < 0.01 indicating significant difference compared to the control.

## 3. Results and Discussion

### 3.1. Protein Extraction and Purification

Purification of the precipitated proteins [21] with Sephadex G-100 gel filtration chromatography (FPLC, Akta, and GE) using Tris HCl (pH 7.5) resulted in the elution of protein fractions designated as F1 eluted at 15 min, F2 at 25 min, and F3 at 45 min detected at 280 nm (Figure 1). The protein concentration in each fraction was determined according to Lowry et al. (1951). The elution volume of the protein fractions was compared with that of standard molecular weight markers of cytochrome c, *α*-lactalbumin, trypsinogen, ovalbumin and bovine serum albumin. The results obtained in this study suggested that plant proteins with molecular masses of 4 KDa–150 KDa can be separated using Sephadex G-100 chromatography [22]. Earlier Etlzer (1985) reported the purification of plant lectins consisting of subunits ranging in molecular mass from 25 KDa to 35 KDa using Sephadex G-100 chromatography [23]. The purified peak fraction eluted at 45 min was found to possess maximum cytotoxic activity and was named as “protein fraction F3” which was then carried on for further analyses.

### 3.2. Reverse Phase-HPLC Analysis

**Figure 2:**
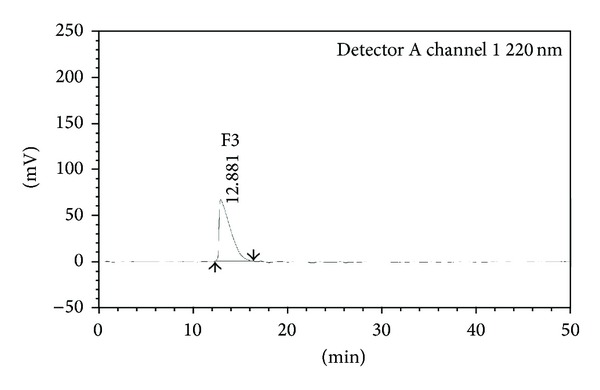
Reverse Phase HPLC of protein fraction F3 isolated from seeds of *B. hispida.*

RP-HPLC of cytotoxic protein fraction F3 showed a single peak (Figure 2) indicating the presence of single protein with a retention time of 12.8 min which was detected at 220 nm. In agreement with this result, it is evident that the protein fraction F3 appeared to have >90% purity which yields a single band with an apparent molecular mass of 35 KDa.

### 3.3. Identification of Protein Fraction (F3) by SDS PAGE

**Figure 3:**
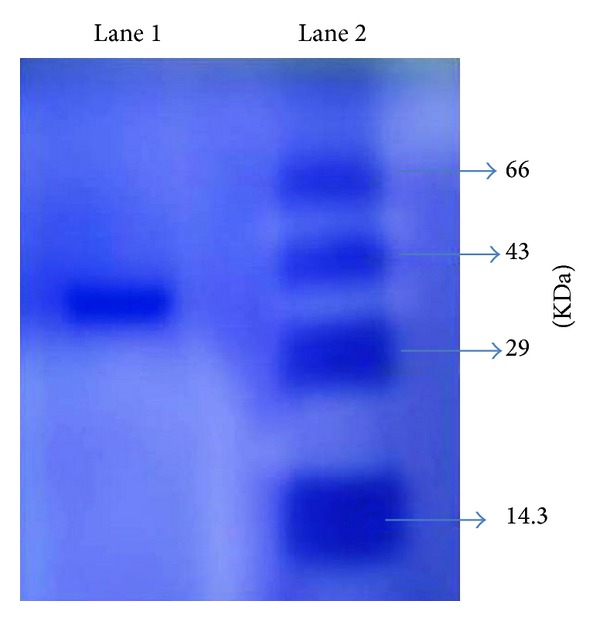
SDS-PAGE analysis of protein fraction F3 purified using Sephadex G-100 chromatogtraphy from the seeds of *B. hispida*. Lane 1: purified protein fraction F3 eluted from FPLC showed a single protein band showing approximate molecular mass of 35 KDa. Lane 2: molecular weight marker (range 14.3 KDa–66 KDa).

The solubilizing denaturing agents such as sodium dodecyl sulphate (SDS) are widely used in the separation of proteins by gel electrophoresis. Identification of approximate mass of the cytotoxic protein fraction F3 was analysed by SDS-PAGE. A single band corresponding to the molecular weight of F3 approximately 35 KDa was observed when compared with the standard molecular weight markers (Figure 3).

### 3.4. Peptide Mass Fingerprinting of Protein Fraction (F3)

**Figure 4:**
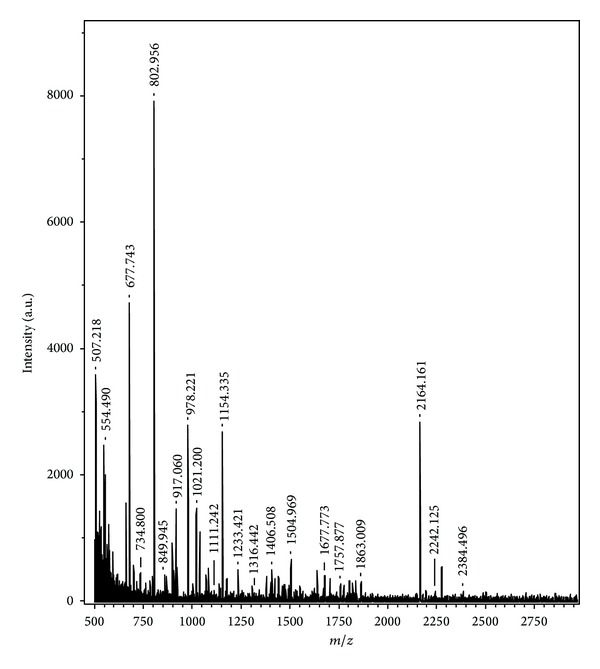
MALDI-TOF/MS spectrum of the protein fraction F3.

Matrix-assisted laser desorption ionization time-of-flight (MALDI-TOF) has become an important tool of choice for large molecular analyses, especially for proteins. The proteome profiling technique by MALDI-TOF-MS provided a broad-base and effective approach for the identification of proteins with biological activity [24, 25]. In the present investigation the gel band of F3 was excised from Coomassie Blue-stained gel and further processed for MALDI-TOF-MS analysis. Each peak in the MALDI-TOF-MS spectrum as given in Figure 4 represents a peptide present in the tryptic digest of protein fraction F3. The size of the peptide ion varies from *m/z *507.21 to 2384.49. The most abundant peptide ions are *m/z *677.74, 802.95, 978.22, 1154.33, and 2164.16 as given in Figure 4. The nominal mass of the active protein F3 was found to be 35166 Da as obtained from peptide mass fingerprinting analysis. Identification of F3 using SWISS-PROT database which contains theoretical tryptic digests of all known proteins revealed the sequence similarity with 1–325 amino acids of 1-aminocyclopropane-1-carboxylatedeaminase (ACCD) of hyperthermophilic archearon, *Pyrococcus horikoshii* OT3 (Box 1). The molecular mass of mapped peptides of ACCD with F3 is given in Table 1. ACCD is a pyridoxal 5′-phosphate dependent enzyme that shows deaminase activity toward ACC, a precursor of plant hormone ethylene. ACCD has been reported to break the cyclopropane ring of ACC to yield *α*-ketobutyrate and ammonia [26].

### 3.5. Cytotoxic Effect of Protein Fractions on Lung (A549) and Cervical (HeLa) Cancer Cells

Cultured cancer cells are valuable reagents not only for rapid screening of potential anticancer agents but also for elucidation of mechanism of their activity. In this study, cytotoxic activity of purified protein fractions was determined using MTT assay on human lung (A549) and cervical (HeLa) cells. The results of the study clearly marked that only F3 fraction exerted more significant cytotoxic activity than F1 and F2 fractions against tested cell lines at concentrations ranging from 10 *μ*g −1000 *μ*g/mL as shown in Tables 2 and 3. Doxorubicin at concentrations ranging from 0.1 *μ*g/mL to 10 *μ*g/mL inhibited the cell proliferation of A549 and HeLa cells and served as a positive control. Untreated A549 and HeLa cells served as cell control exhibiting 100% cell viability (Tables 2 and 3). Morphological changes consistent with apoptotic cell death were seen in A549 and HeLa cells treated with F3 isolated from seeds of *B. hispida *as evidenced by microscopic observations shown in Supplementary Material available online at http://dx.doi.org/10.1155/2014/179836. Cell shrinkage and rounding, were observed in A549 cells indicating 59% cell inhibition, when treated with F3 at the concentration of 10 *μ*g/mL as given in Table 2. Increased inhibition of cell proliferation was observed in A549 cells treated with F3 at concentrations of 30 *μ*g/mL showing 78% inhibition and 100–1000 *μ*g/mL of F3 showing >90% inhibition of cell proliferation in A549 cells as evidenced in the Supplementary Material. Further the results of MTT assay also showed that protein fraction F3 effectively suppressed the proliferation of HeLa cells with 61.38% inhibition (Table 3) at the concentration of 10 *μ*g/mL depicting chromatin condensation (pyknosis) in the nucleus of cells undergoing apoptosis as shown in the Supplementary Material. Similar morphological alterations associated with pyknosis and karyorrhexis (nuclear fragmentation) were observed in cancer cells treated with cytotoxic proteins isolated from the various plant sources [27, 28]. Apoptosis is an important homeostatic mechanism that balances cell division and cell death for maintaining the appropriate cell number in the body. Induction of apoptosis in cancer cells is one of the strategies for anticancer drug development [29, 30].

### 3.6. Effect of F3 on Cell Cycle of A549 and HeLa Cells

Disturbance of the cancer cell cycle is one of therapeutic targets for development of new anticancer drugs [31]. The distribution of cell population in each phase of cell cycle was examined by flow cytometry. Our results revealed that apoptosis induced by F3 fraction was not triggered at a specific phase of the cell cycle. However, F3 fraction increased the number of apoptotic cells of sub-G0 population in a time-dependent manner. Cell cycle analysis performed on A549 cells treated with F3 at a concentration of 1000 *μ*g/mL for different time periods revealed interesting observations. Marked changes in the sub-G0 population (hypodiploid cells) of A549 cells were observed recording 2.11% and 2.71% increases (Table 4) at 24 and 48 h as given in the Supplementary Material. A successful anticancer compound should kill or incapacitate cancer cells without causing excessive damage to normal cells. It is evident that there is no growth in sub-G0 phase of cell cycle in control cells of both the A549 and HeLa cells which indicated the nonapoptotic population. 

The most pronounced cytotoxic effect was obtained with HeLa cells treated with F3 at concentration of 1000 *μ*g/mL, leading to increased cell population in sub-G0 phase as 3.07% and 3.42% (Table 5) at 24 and 48 h, indicating increased apoptotic-associated chromatin degradation in HeLa cells as shown in the Supplementary Material. Similar observations were noted with nebrodeolysin, isolated from *Pleurotus nebrodensis* which induced apoptosis in HeLa cancer cells [32]. The protein fraction isolated from the leaves of *Mirabilis jalapa *L. was cytotoxic to HeLa cells causing DNA fragmentation and apoptosis [33]. Reports have demonstrated that the toxin abrin, a type of ribosome inactivating protein, induced cell cycle arrest and apoptotic cell death in HeLa cells [34]. Other proteins isolated from different plant sources also have been found to exhibit antiproliferation action involving cell cycle arrest in HeLa cells [35, 36].

## 4. Conclusion

This study is the first report of identification of the cytotoxic protein with molecular mass of 35 KDa from the seeds of *Borreria hispida *showing sequence similarity with 1–325 amino acids of 1-aminocyclopropane-1-carboxylate deaminase (ACCD) of hyperthermophilic archearon, *Pyrococcus horikoshii* OT3. The cytotoxic protein F3 is found to exhibit growth inhibition (*in vitro *cytotoxicity assay) and cell cycle arrest in sub-G0 population of human lung (A549) and cervical (HeLa) cancer cells as examined by flow cytometer.

## Supplementary Material

Figure 1: The active protein fraction – F3 at concentrations ranging from 10-1000µg/mL exhibited increased cytotoxic activity in A549 cells, evaluated by MTT assay associated with chromatin condensation in the tested cell line as evidenced in the microscopic observations. Arrow marks indicate the presence of apoptotic cells.
Figure 2: Potent cytotoxic activity was noted in HeLa cells treated with 10-1000µg/mL of protein fraction – F3, analysed by MTT assay, resulted in the fragmentation of cell nucleus in the tested cell line as depicted in the micrographs. Arrow marks indicate the presence of apoptotic cells.
Figure 3: Flow cytometry analysis of four phases of cell cycle (SubG0, G1, S, G2 and M) in A549 cells treated with protein fraction – F3 at a concentration of 1000µg/mL depicted the induction of apoptosis in A549 cells at SubG0-G1 phase as compared to the non apoptotic population in cell control (untreated A549 cells).
Figure 4: HeLa cell cycle analysis (SubG0, G1, S, G2 and M phases) by Flow cytometry revealed the apoptotic effect of protein fraction – F3 at a concentration of 1000µg/mL, showing increased number of apoptotic cells at SubG0-G1 phase in HeLa cells and absence of apoptosis in control (untreated HeLa cells).

## Figures and Tables

**Box 1:**
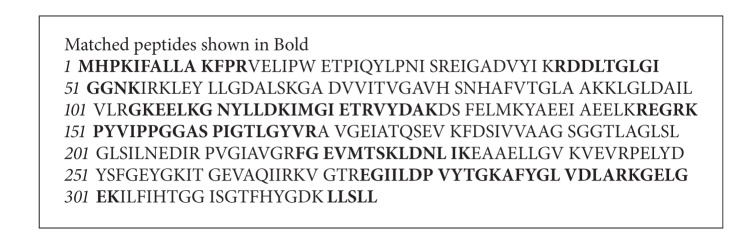
Peptide mass fingerprinting analysis of protein fraction F3 showed sequence similarity (shown in bold) with amino acid sequences of 1-aminocyclopropane-1-carboxylate deaminase (1–325) of *Pyrococcus horikoshii* OT3 identified from SWISS-PROT database.

**Table 1:** The molecular mass of peptides and their aminoacid sequences (1–325 aminoacids) of 1-aminocyclopropane-1-carboxylate deaminase of *Pyrococcus horikoshii* OT3.

Start–end	Observed	Mr (expt)	Mr (calc)	Delta	Miss	Sequence
1–4	511.2710	510.2637	511.2577	−0.9940	0	**-.MHPK.I**
1–4	512.2440	511.2367	511.2577	−0.0210	0	**-.MHPK.I**
1–4	513.2960	512.2887	511.2577	1.0310	0	**-.MHPK.I**
1–4	527.3730	526.3657	527.2526	−0.8869	0	**-.MHPK.I** (M)
1–4	529.3150	528.3077	527.2526	1.0551	0	**-.MHPK.I** (M)
1–11	1284.54	1283.533	1283.7424	−0.2086	1	**-.MHPKIFALLAK.F** (M)
2–11	1138.289	1137.2817	1136.7070	0.5748	1	**M.HPKIFALLAK.F**
5–14	1176.308	1175.3007	1174.7226	0.5781	1	**K.IFALLAKFPR.V**
42–54	1316.44	1315.4347	1314.6892	0.7455	1	**K.RDDLTGLGIGGNK.I**
104–109	703.71	702.7087	702.3912	0.3175	1	**R.GKEELK.G**
106–109	519.355	518.3477	517.2748	1.0730	0	**K.EELK.G**
110–123	1639.799	1638.7917	1637.8447	0.9471	1	**K.GNYLLDKIMGIETR.V** (M)
124–128	594.397	593.3897	594.3013	−0.9116	0	**R.VYDAK.D**
146–149	516.345	515.3377	516.2768	−0.9391	1	**K.REGR.K**
146–149	517.331	516.3237	516.2768	0.0469	1	**K.REGR.K**
147–169	2384.49	2383.4887	2383.3012	0.1875	1	**R.EGRKPYVIPPGGASPIGTLGYVR.A**
219–226	898.02	897.0167	897.4266	−0.4099	0	**R.FGEVMTSK.L**
219–232	1610.62	1609.6177	1609.8385	−0.2208	1	**R.FGEVMTSKLDNLIK.E** (M)
274–285	1304.53	1303.5247	1303.7024	−0.1776	0	**R.EGIILDPVYTGK.A**
286–296	1252.11	1251.1087	1251.6975	−0.5888	1	**K.AFYGLVDLARK.G**
296–302	760.8440	759.8367	759.4126	0.4241	1	**R.KGELGEK.I**
321–325	557.343	556.3357	557.3788	−1.0431	0	**K.LLSLL.-**

**Table 2:** Percentage inhibition of A549 cells treated with different concentrations of F3 isolated from seeds of *B. hispida.* Cell viability was measured by MTT assay and the values are expressed as mean ± SEM.

Concentration (µg/mL)	F3 (protein fraction)	Doxorubicin	Cell control
10	59.80 ± 6.05****		
30	78.06 ± 4.76****		
100	89.00 ± 4.70***		
300	89.76 ± 4.87***		
1000	88.71 ± 5.41**		
0.1		52.83 ± 1.15****	
0.3		70.95 ± 2.82****	
1		85.17 ± 1.38***	
3		87.35 ± 4.33***	
10		99.11 ± 0.53***	
			100

****P < 0.0001, ***P < 0.001, and **P < 0.01 indicate significant difference compared to the control. Values are analyzed by using Dunnett's Multiple Comparison Test.

**Table 3:** Percentage inhibition of HeLa cells treated with different concentrations of F3 isolated from seeds of *B. hispida.* Cell viability was measured by MTT assay and the values are expressed as mean ± SEM.

Concentration (µg/mL)	F3 (protein fraction)	Doxorubicin	Cell control
10	61.38 ± 2.70****		
30	68.06 ± 5.17****		
100	99.46 ± 0.50***		
300	98.80 ± 1.94***		
1000	98.92 ± 1.14**		
0.1		20.39 ± 0.44****	
0.3		36.86 ± 1.57****	
1		62.10 ± 1.66***	
3		84.89 ± 2.98***	
10		99.54 ± 0.46***	
			100

****P < 0.0001, ***P < 0.001, and **P < 0.01 indicate significant difference compared to the control. Values are analyzed by using Dunnett's Multiple Comparison Test.

**Table 4:** Cell cycle analysis of A549 cells treated with protein fraction F3 (1000 µg/mL) from the seeds of *Borreria hispida. *

S. number	Phases of cell cycle	Control cell count (%)		Treated cell count (%)	
		24 h	48 h	24 h	48 h
1	Sub-G0-G1	0	0	2.11	2.71
2	G0-G1	61.78	62.37	51.3	46.5
3	S	20.26	10.7	15.5	14.7
4	G2-M	6.8	6.6	7.21	6.5

**Table 5:** Cell cyle analysis of HeLa cells treated with protein fraction F3 (1000 µg) from the seeds of *Borreria hispida. *

S. number	Phases of cell cycle	Control cell count (%)		Treated cell count (%)	
		24 h	48 h	24 h	48 h
1	Sub-G0-G1	0	0	3.07	3.42
2	G0-G1	61.5	55.43	59.86	55.86
3	S	20.44	19.97	17.04	17.03
4	G2-M	9.42	8.89	6.13	5.38
