# Supplementary material for: Induction of Apoptotic Effects of Antiproliferative Protein from the Seeds of Borreria hispida on Lung Cancer (A549) and Cervical Cancer (HeLa) Cell Lines
Source: Biomed Res Int. 2014 Jan 30;2014:179836. doi: 10.1155/2014/179836 (PMC3925513; doi:10.1155/2014/179836)

Supplementary File

1. Photomicrographs (40x) of A549 cells treated with different concentrations of F3 isolated from seeds of *B. Hispida*.Morphological changes are indicated with arrow marks.

.

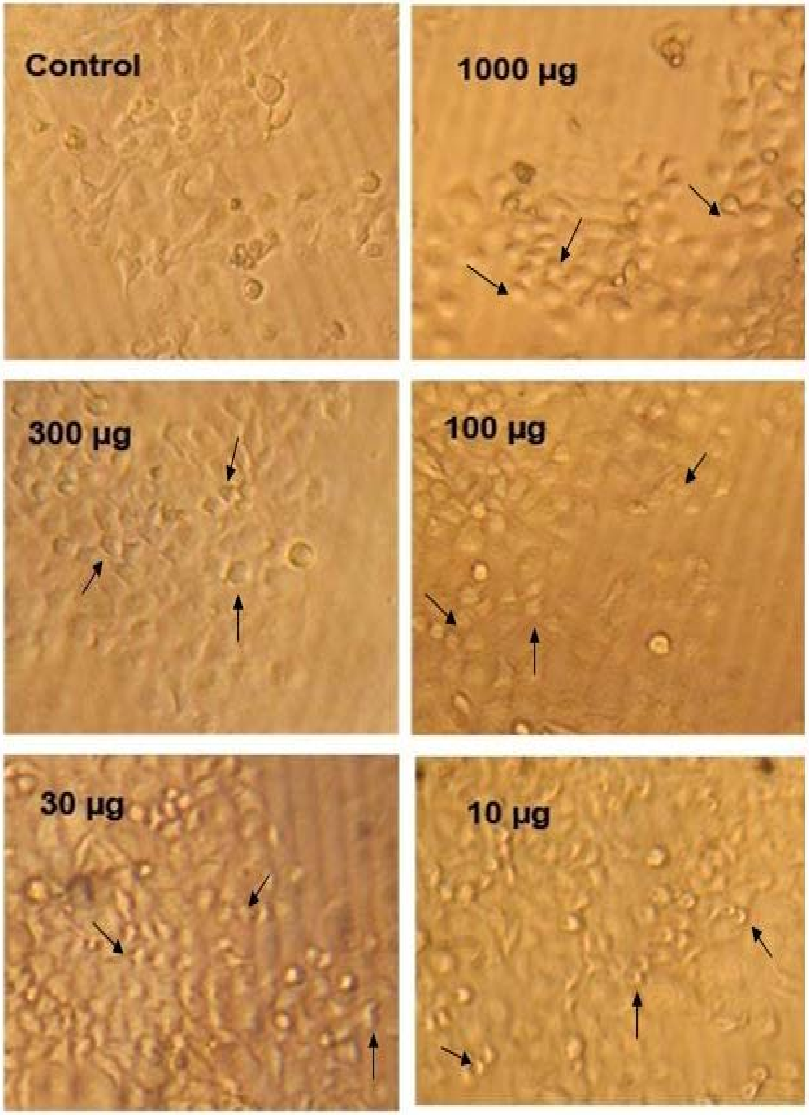

2. Photomicrographs (40x) of HeLa cells treated with different concentrations of F3 isolated from seeds of *B. hispida*. Morphological changes are indicated with arrow marks.

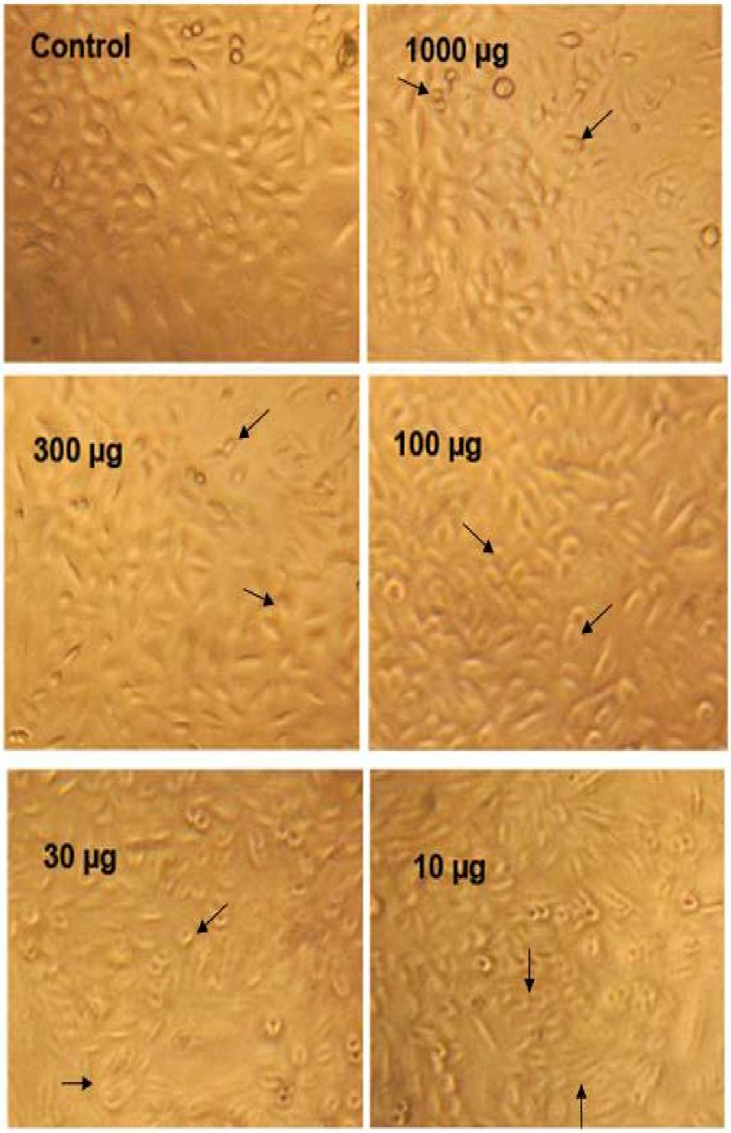

3. Effect of the Protein Fraction F3 on the cell cycle of A549 cells for 24 and 48h

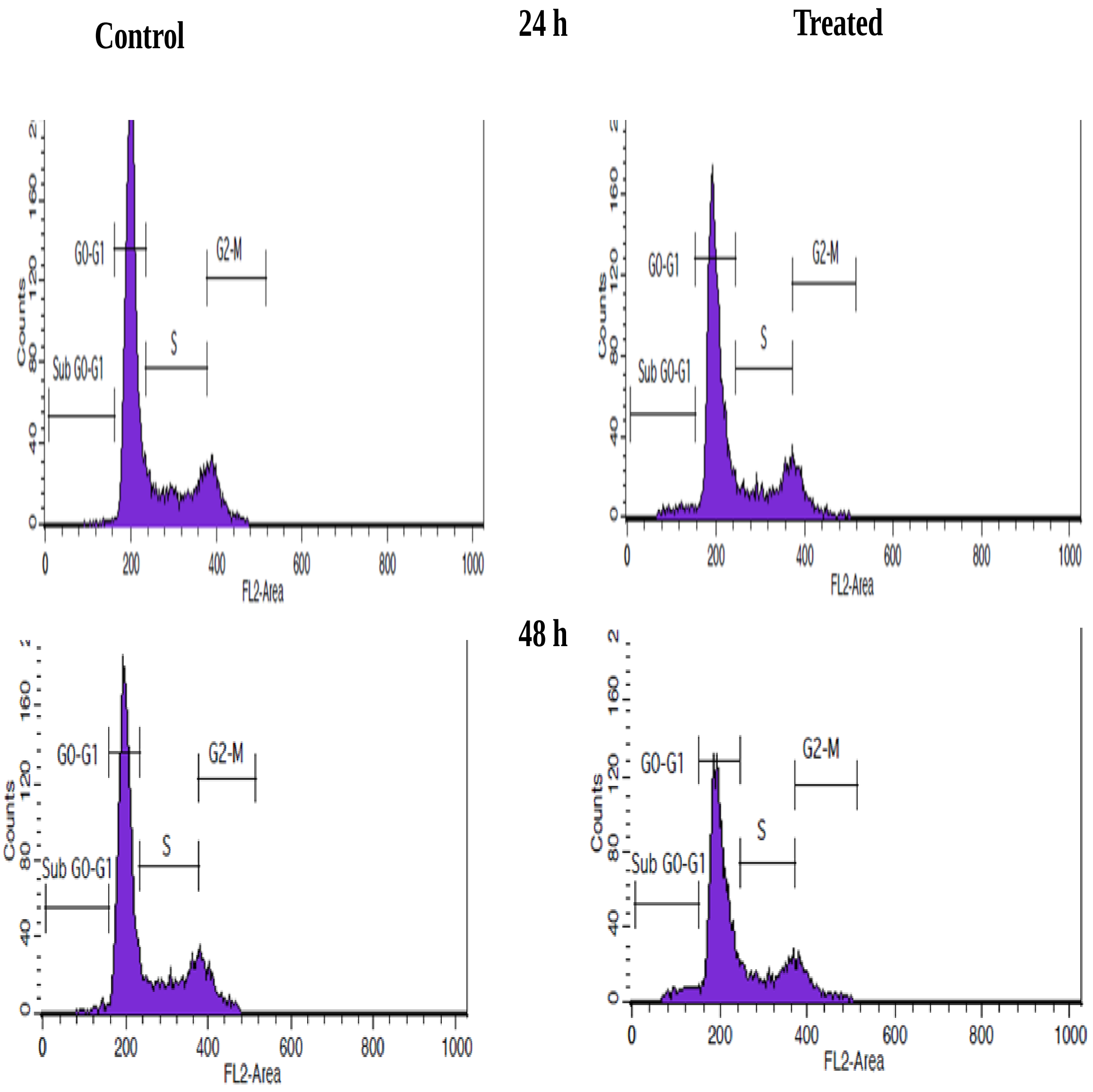

4. Effect of protein fraction F3 on the cell cycle of HeLa cells

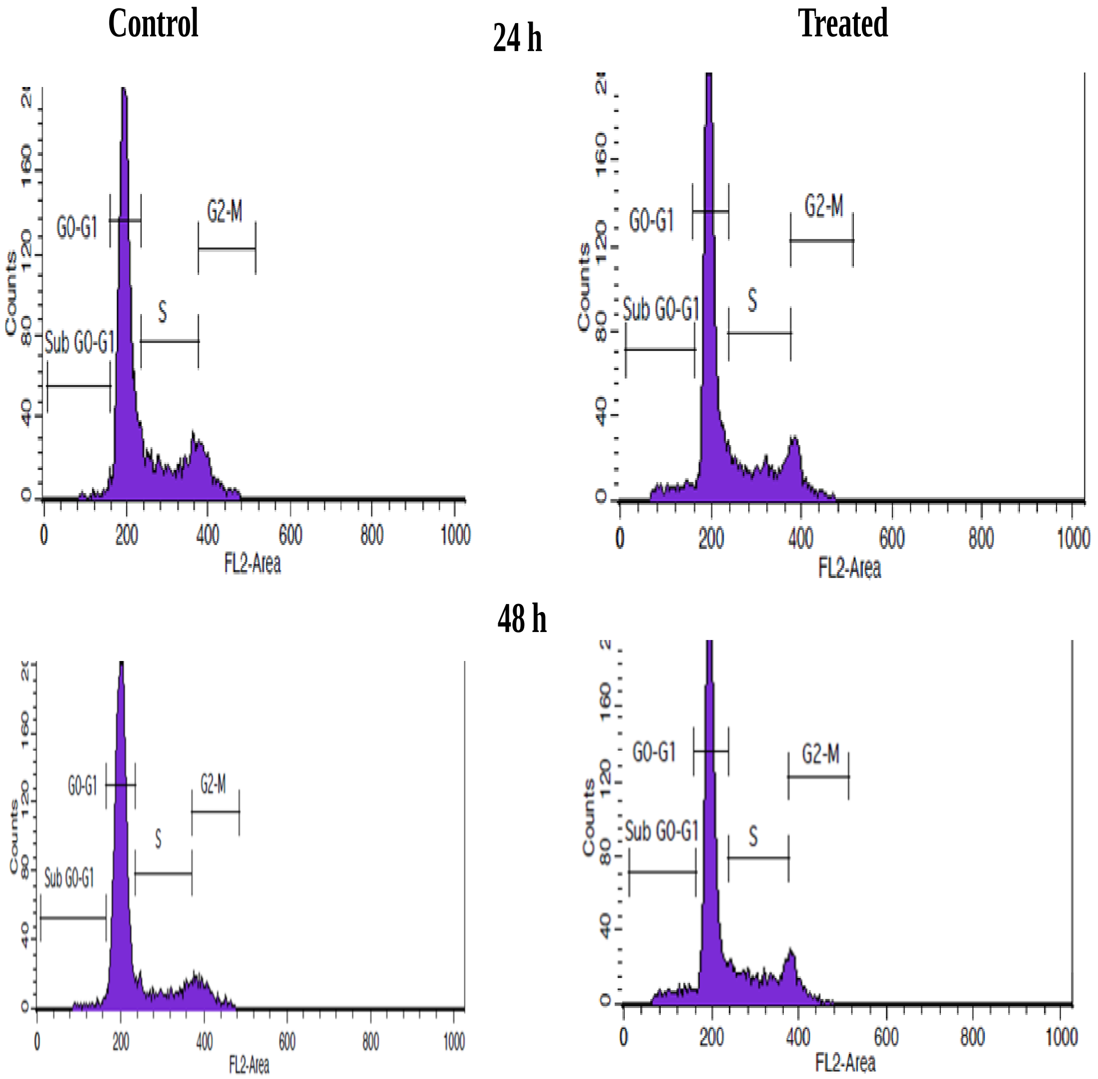

Supplement: Supplementary file 1 — Figure 1: The active protein fraction – F3 at concentrations ranging from 10-1000µg/mL exhibited increased cytotoxic activity in A549 cells, evaluated by MTT assay associated with chromatin condensation in the tested cell line as evidenced in the microscopic observations. Arrow marks indicate the presence of apoptotic cells. Figure 2: Potent cytotoxic activity was noted in HeLa cells treated with 10-1000µg/mL of protein fraction – F3, analysed by MTT assay, resulted in the fragmentation of cell nucleus in the tested cell line as depicted in the micrographs. Arrow marks indicate the presence of apoptotic cells. Figure 3: Flow cytometry analysis of four phases of cell cycle (SubG0, G1, S, G2 and M) in A549 cells treated with protein fraction – F3 at a concentration of 1000µg/mL depicted the induction of apoptosis in A549 cells at SubG0-G1 phase as compared to the non apoptotic population in cell control (untreated A549 cells). Figure 4: HeLa cell cycle analysis (SubG0, G1, S, G2 and M phases) by Flow cytometry revealed the apoptotic effect of protein fraction – F3 at a concentration of 1000µg/mL, showing increased number of apoptotic cells at SubG0-G1 phase in HeLa cells and absence of apoptosis in control (untreated HeLa cells). [file 179836.f1.pdf]
